# Ultrasound-based artificial intelligence for predicting cervical lymph node metastasis in papillary thyroid cancer: a systematic review and meta-analysis

**DOI:** 10.3389/fendo.2025.1570811

**Published:** 2025-06-10

**Authors:** Xi Wang, Yiting Qi, Xin Zhang, Fang Liu, Jia Li

**Affiliations:** ^1^ Department of Nursing, Zhuhai Campus of Zunyi Medical University, Guangdong, China; ^2^ Department of Ultrasound Imaging, Zhuhai People’s Hospital, Zhuhai, Guangdong, China; ^3^ Department of Nursing, Kiang Wu Nursing College of Macau, Macau, China

**Keywords:** artificial intelligence, ultrasonography, cervical lymph node metastasis, papillary thyroid cancer, meta-analysis

## Abstract

**Objective:**

This meta-analysis aims to evaluate the diagnostic performance of ultrasound (US)-based artificial intelligence (AI) in assessing cervical lymph node metastasis (CLNM) in patients with papillary thyroid carcinoma (PTC).

**Methods:**

A comprehensive literature search was conducted in PubMed, Embase, Web of Science, and the Cochrane Library to identify relevant studies published up to November 19, 2024. Studies focused on the diagnostic performance of AI in the detection of CLNM of PTC were included. A bivariate random-effects model was used to calculate the pooled sensitivity and specificity, both with 95% confidence intervals (CI). The I^2^ statistic was used to assess heterogeneity among studies.

**Results:**

Among the 593 studies identified, 27 studies were included (involving over 23,170 patients or images). For the internal validation set, the pooled sensitivity, specificity, and AUC for detecting CLNM of PTC were 0.80 (95% CI: 0.75–0.84), 0.83 (95% CI: 0.80–0.87), and 0.89 (95% CI: 0.86–0.91), respectively. For the external validation set, the pooled sensitivity, specificity, and AUC were 0.77 (95% CI: 0.49–0.92), 0.82 (95% CI: 0.75–0.88), and 0.86 (95% CI: 0.83–0.89), respectively. For US physicians, the overall sensitivity, specificity, and AUC for detecting CLNM were 0.51 (95% CI: 0.38–0.64), 0.84 (95% CI: 0.76–0.89), and 0.77 (95% CI: 0.73–0.81), respectively.

**Conclusion:**

US-based AI demonstrates higher diagnostic performance than US physicians. However, the high heterogeneity among studies and the limited number of externally validated studies constrain the generalizability of these findings, and further research on external validation datasets is needed to confirm the results and assess their practical clinical value.

**Systematic review registration:**

https://www.crd.york.ac.uk/PROSPERO/view/CRD42024625725, identifier CRD42024625725.

## Introduction

Papillary thyroid carcinoma (PTC) is the most common malignant thyroid tumor, with a steadily increasing global incidence, though its mortality rate remains relatively low (1). Approximately 30% to 80% of PTC patients experience lymph node metastasis (LNM), with cervical lymph node metastasis (CLNM) occurring in about 49% of these LNM-positive patients (2, 3). CLNM is a major risk factor for recurrence and reduced survival, often requiring aggressive surgical interventions, such as extensive lymph node dissection, which carry higher risks of complications (4). Accurate and timely detection of CLNM is therefore critical, as it directly influences treatment strategies and improves patient outcomes.

Traditional imaging modalities, including ultrasound (US), computed tomography (CT), magnetic resonance imaging (MRI), and positron emission tomography-computed tomography (PET-CT), are widely used for evaluating CLNM of PTC (5). Among these, US is the first-line tool due to its non-invasive nature, real-time imaging capabilities, and high spatial resolution (6). However, its diagnostic accuracy is highly operator-dependent, leading to inconsistent results (7). In contrast, CT and MRI offer more detailed anatomical insights but have low sensitivity in identifying small metastatic lymph nodes (<2–3 mm), increasing the risk of missed diagnoses (8, 9). Moreover, these methods often rely on qualitative or semi-quantitative assessments, such as lymph node size and morphology, while neglecting quantitative features like texture, density, and signal intensity, which may be critical for predicting CLNM (10). These limitations highlight the need for more advanced diagnostic tools.

Artificial intelligence (AI) offers promising opportunities to improve the diagnostic performance of US in detecting CLNM. AI algorithms, particularly those based on machine learning and deep learning, can analyze complex imaging data and extract subtle features beyond human perception (11, 12). These algorithms process high-dimensional data and identify patterns that traditional methods may overlook. However, the diagnostic performance of AI remains inconsistent across studies (13, 14), and its comparative performance versus experienced US physicians has not been fully established, raising questions about its integration into routine clinical practice (15).

This meta-analysis aims to systematically evaluate the performance of US-based AI and its relative effectiveness compared to US physicians in detecting CLNM of PTC, providing a comprehensive assessment of its diagnostic capabilities and potential impact on clinical practice.

## Methods

The meta-analysis was carried out strictly following the Preferred Reporting Items for Systematic Reviews and Meta-Analyses for Diagnostic Test Accuracy (PRISMA-DTA) guidelines (16). Moreover, the protocol of this study has been registered with the PROSPERO (CRD42024625725).

### Search strategy

A comprehensive search across PubMed, Embase, Web of Science, and Cochrane Library, with cutoff date of November 19, 2024. The search strategy included three groups of keywords: the first group related to AI (e.g., artificial intelligence, machine learning, deep learning), the second group related to diseases (e.g., lymphatic metastasis, lymph node metastasis), the third group related to target condition (e.g., thyroid neoplasms, thyroid carcinoma). We employed a combination of Medical Subject Headings (MeSH) and keywords (see **Supplementary Table S1**). Only studies published in English with full texts were included. Additionally, we manually searched the reference lists of selected studies to identify any potentially missed relevant articles. To ensure no recent studies were overlooked, we repeated the literature search on December 21, 2024.

### Inclusion and exclusion criteria

Studies were carefully selected based on the PICOS framework. Population (P): Participants included patients diagnosed with PTC who required evaluation for CLNM. Intervention (I): AI models based on US images. Comparison (C): Either without a control group or compared with experienced ultrasound physicians. Outcome (O): The primary outcomes of interest included sensitivity, specificity, and area under the receiver operating characteristic curve (AUC). Study design (S): Both retrospective and prospective study designs were included.

We excluded animal studies and non-original research articles, including reviews, case reports, conference abstracts, meta-analyses, and letters to the editor. In addition, non-English full-text articles were excluded. Studies that did not meet these criteria were excluded from further analysis.

### Quality assessment

We employed a modified version of the Quality Assessment of Diagnostic Performance Studies Revised (QUADAS-2-Revised tool) tool (17) to comprehensively evaluate the methodological quality of included studies. The adaptation involved replacing certain non-relevant criteria with more pertinent standards from the Prediction Model Risk of Bias Assessment tool, accounting for potential sources of bias arising from variations in research design and implementation.

The QUADAS-2-Revised tool assessed four critical domains: participants, index test (AI algorithm), reference standard, and analysis. The detail criteria were shown in **Supplementary Table S2**. Two independent reviewers systematically evaluated each domain’s risk of bias, with a particular focus on applicability in the first three domains. Divergent assessments were resolved through collaborative discussion.

### Data extraction

Two reviewers independently evaluated the eligibility of studies and extracted data. In cases of disagreement, a third reviewer acted as an arbitrator to facilitate consensus. The extracted data included the first author’s name, publication year, country of study origin, study type, AI methods, selected AI algorithms, AI models, and patient-related data.

Since most studies did not report diagnostic contingency tables, we employed two methods to determine the diagnostic 2×2 table: 1) using sensitivity, specificity, the number of true positives determined by the reference standard, and the total number of cases; 2) through receiver operating characteristic (ROC) curve analysis, extracting sensitivity and specificity based on the optimal Youden index.

### Outcome measures

The primary outcome measures included sensitivity, specificity, and area under the curve (AUC) for internal validation sets, external validation sets, and radiologists. Sensitivity (also known as recall or true positive rate) measures the probability that the AI model correctly identifies true positive cases, calculated as TP/(TP+FN). Specificity (also known as true negative rate) reflects the probability that the AI model correctly identifies healthy cases, calculated as TN/(TN+FP). AUC represents the area under the ROC curve, serving as a comprehensive measure of the model’s ability to distinguish between positive and negative cases. We extracted AI diagnostic performance data from internal validation sets, external validation sets, and US physicians, including only the models with optimal diagnostic performance (highest AUC values).

### Statistical analysis

We summarized the overall sensitivity and specificity of AI analyses predicting CLNM of PTC using a bivariate random effects model for internal validation sets, external validation sets, and clinical diagnoses (18). A forest plot was created to visually represent the pooled sensitivity and specificity. Moreover, a summary receiver operating characteristic (SROC) curve was constructed to illustrate the overall sensitivity and specificity estimates along with their 95% confidence intervals (CI) and prediction intervals. Additionally, a Fagan plot was generated to evaluate the clinical applicability.

Heterogeneity among the included studies was assessed using the I^2^ statistic, with I^2^ values of 25%, 50%, and 75% indicating low, moderate, and high heterogeneity, respectively (19). For internal validation sets (greater than 10 studies), meta-regression analysis was conducted when significant heterogeneity was present (I^2^>50%) to explore potential sources of heterogeneity. The variables for meta-regression included US techniques (B-mode US or multimodal US), AI algorithms, AI models, data analysis types, and the location of CLNM. Furthermore, subgroup analyses were conducted for these variables to assess differences between subgroups. We also used the Z-test to compare the outcome differences between the internal validation sets and US physicians (20). Publication bias was assessed using Deeks’ funnel plot. Statistical analyses were primarily conducted using the Midas and Metadta programs in STATA version 15.1. The risk of bias assessment for study quality was performed using RevMan 5.4 (Cochrane Collaboration). A *P*-value of <0.05 was defined as statistically significant.

## Results

### Study selection

**Figure 1:**
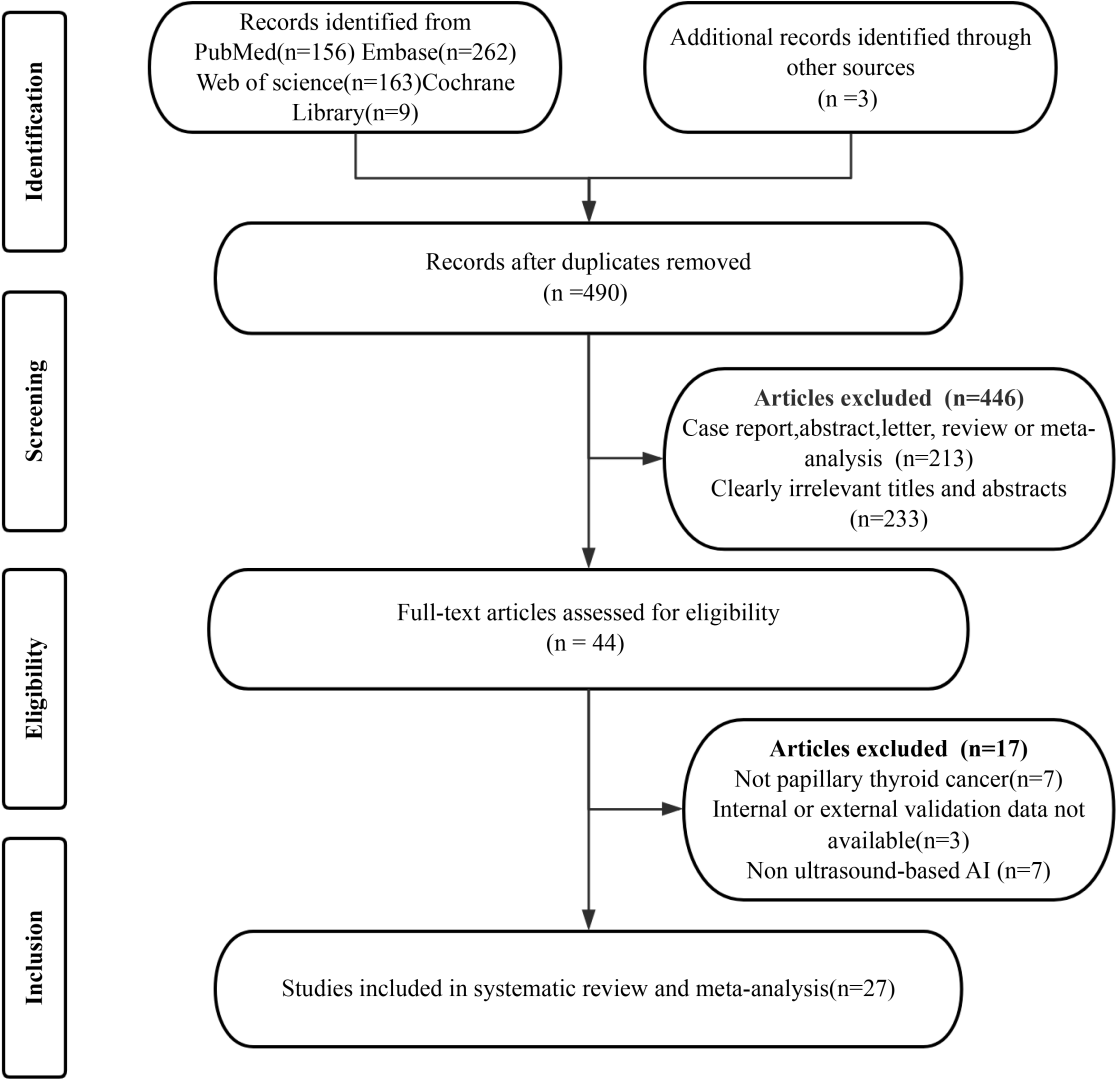
PRISMA flow diagram illustrating the study selection process.

The initial database search yielded 593 potentially relevant articles. After removing 103 duplicates, 490 unique articles proceeded to preliminary screening. Following a rigorous application of the inclusion criteria, 446 articles were excluded. After a detailed full-text review, 17 studies were further excluded, including seven studies for not being PTC, three studies due to internal or external validation data being unavailable, and seven studies for being non-US-based AI. Ultimately, 27 studies that met the criteria for evaluating AI diagnostic performance were included in the meta-analysis (2, 13, 21–45). The literature selection method is comprehensively outlined in accordance with the standardized Preferred Reporting Items for Systematic Reviews and Meta-Analyses (PRISMA) flow diagram, as shown in **Figure 1**.

### Study description and quality assessment

A total of 27 eligible studies were identified, with the internal validation set comprising all 27 studies and a total of 6,366 patients (range: 50-1,013), while the external validation set included 4 studies with a total of 1,592 patients (range: 95-881). 13 articles provided diagnostic data from US clinicians. One study was prospective, while 26 were retrospective design. Of the studies, 24 used pathology as the gold standard, and three utilized fine needle aspiration (FNA) as the gold standard. The most common modeling methods were logistic regression (LR) (12/27, 44%), convolutional neural network (CNN) (7/27, 26%), and support vector machine (SVM) (2/27, 7%). The characteristics of the studies and patients are summarized in **Tables 1** and **2**.

**Table 1:** Study and patient characteristics of the included studies.

Author	Year	Country	Study design	Imaging modality	Location of cervical lymph node metastasis	Analysis	Reference standard	Patients/lesions per set			No. of LNM+ patients/lesions/
								Training	Internal validation	External validation	
Agyekum et al. (2)	2022	China	Retro	B-mode	Central	Patient-based	Pathology	143	62	NR	Training: 74 Internal validation: 33
Chang et al. (21)	2023	China	Retro	B-mode	Central	Patient-based	Pathology	2114	906	339	Training: 1063 Internal validation: 460 External validation:162
Chen et al. (22)	2021	China	Retro	B-mode	Central	Patient-based	Pathology	634	272	NR	Training: 228 Internal validation: 94
Dai et al. (23)	2023	China	Retro	CDU&EG	Central	Patient-based	Pathology	348	150	NR	Training: 167 Internal validation: 74
Gao et al. (13)	2024	China	Retro	B-mode	Central	Patient-based	Pathology	460	153	NR	Training: 228 Internal validation: 76
Guang et al. (24)	2023	China	Retro	B-mode	Central& Lateral	Patient-based	Pathology	196	50	NR	Training: 100 Internal validation: 26
Huang et al. (25)	2021	China	Retro	EG&CDU	Central	Patient-based	Pathology	439	220	NR	Training: 160 Internal validation: 77
Jia et al. (26)	2024	China	Retro	SWE&CEUS	Central	Patient-based	Pathology	NR	126	NR	Internal validation: 59
Jiang et al. (27)	2020	China	Retro	SWE&CDU	Central& Lateral	Patient-based	Pathology	147	90	NR	Training: 75 Internal validation: 38
Jiang et al. (28)	2023	China	Retro	CEUS	NR	Patient-based	Pathology	148	63	NR	Training: 59 Internal validation: 29
Qian et al. (29)	2024	China	Retro	DUV	NR	Patient-based	Pathology	233	78	NR	Training: 108 Internal validation: 30
Shi et al. (30)	2022	China	Retro	B-mode	Central	Patient-based	Pathology	469	118	NR	Training: 121 Internal validation: 32
Tong et al. (31)	2022	China	Retro	B-mode	Central& Lateral	Patient-based	Pathology	300	143	277	Training: 104 Internal validation: 47 External validation:112
Tong et al. (32)	2021	China	Retro	B-mode	Lateral	Patient-based	Pathology	600	286	NR	Training: 55 Internal validation: 31
Wang et al. (33)	2024	China	Pro	SWE	NR	Lesion-based	FNA	NR	84	NR	Internal validation:36
Wei et al. (34)	2023	China	Retro	CEUS	NR	Patient-based	Pathology	282	141	NR	Training: 138 Internal validation: 68
Wen et al. (35)	2022	China	Retro	B-mode	Central	Patient-based	Pathology	353	68	NR	Training: 185 Internal validation: 35
Wu et al. (36)	2024	China	Retro	EG	Central	Patient-based	FNA	142	62	NR	Training: 75 Internal validation: 27
Park et al. (37)	2020	South Korea	Retro	B-mode	Lateral	Patient-based	Pathology	400	368	NR	Training: 83 Internal validation: 100
Yan et al. (38)	2023	China	Retro	B-mode	Central	Lesion-based	Pathology	212	83	NR	Training: 115 Internal validation: 45
Yao et al. (39)	2022	China	Retro	B-mode	NR	Patient-based	Pathology	5129	903	NR	Training: 2165 Internal validation: 553
Yu et al. (40)	2020	China	Retro	B-mode	Central	Patient-based	Pathology	NR	1013	368,513	Internal validation: 403 External validation: 217,218
Yuan et al. (41)	2024	China	Retro	B-mode	Lateral	Lesion-based	FNA	655	206	NR	Training: 327 Internal validation: 110
Zhang et al. (42)	2025	China	Retro	B-mode	Central	Patient-based	Pathology	340	83	95	Training: 185 Internal validation: 47 External validation:47
Zhang et al. (43)	2023	China	Retro	CDU	NR	Patient-based	Pathology	451	194	NR	Training: 67 Internal validation: 35
Zhou et al. (44)	2022	China	Retro	B-mode	Central	Patient-based	Pathology	608	326	NR	Training: 182 Internal validation: 113
Zhu et al. (45)	2023	China	Retro	B-mode	Central& Lateral	Lesion-based	Pathology	282	118	NR	Training: 117 Internal validation: 38

Retro, retrospective; Pro, prospective; NR, not report; FNA, fine needle aspiration; B-mode, B mode ultrasound; CDU, color doppler ultrasound; EG, elastography; CEUS, contrast-enhanced ultrasound; SWE, shear wave elastography; DUV, dynamic ultrasound video.

**Table 2:** Technical aspects of included studies.

Author	Year	AI method	Optimal AI Algorithm	AI Mode	Interval validation sets				External validation sets				Ultrasound physician			
					TP	FP	FN	TN	TP	FP	FN	TN	TP	FP	FN	TN
Agyekum et al. (2)	2022	Machine learning	LDA	Ultrasound&clinical model	20	8	13	21	NR	NR	NR	NR	49	39	49	68
Chang et al. (21)	2023	Deep learning	CNN	Ultrasound&clinical model	182	104	278	342	59	41	103	136	169,59	34,15	291,103	412,162
Chen et al. (22)	2021	Deep learning	CNN	Ultrasound-based model	81	33	13	145	NR	NR	NR	NR	NR	NR	NR	NR
Dai et al. (23)	2023	Machine learning	SVM	Ultrasound&clinical model	59	8	15	68	NR	NR	NR	NR	NR	NR	NR	NR
Gao et al. (13)	2024	Deep learning	CNN	Ultrasound&clinical model	55	14	21	63	NR	NR	NR	NR	32	23	44	54
Guang et al. (24)	2023	Deep Learning	CNN	Ultrasound-based model	21	4	5	20	NR	NR	NR	NR	61	15	97	135
Huang et al. (25)	2021	Machine learning	LR	Ultrasound&clinical model	60	38	17	105	NR	NR	NR	NR	NR	NR	NR	NR
Jiang et al. (27)	2020	Machine learning	LR	Ultrasound&clinical model	33	14	5	38	NR	NR	NR	NR	41	19	72	105
Jiang et al. (28)	2023	Machine learning	LR	Ultrasound&clinical model	24	9	5	25	NR	NR	NR	NR	NR	NR	NR	NR
Qian et al. (29)	2024	Deep Learning	CNN	Ultrasound-based model	26	6	4	42	NR	NR	NR	NR	NR	NR	NR	NR
Jia et al. (26)	2024	Machine learning	SVM	Ultrasound-based model	53	18	6	49	NR	NR	NR	NR	NR	NR	NR	NR
Shi et al. (30)	2022	Machine Learning	XGBoost	Ultrasound&clinical model	28	12	4	74	NR	NR	NR	NR	NR	NR	NR	NR
Tong et al. (31)	2022	Machine Learning	LR	Ultrasound&clinical model	39	17	8	79	80	21	32	144	23,59	9,24	24,53	87,141
Tong et al. (32)	2021	Machine Learning	LR	Ultrasound&clinical model	25	14	6	241	NR	NR	NR	NR	22	31	9	224
Wang et al.	2024	Machine Learning	Fisher	Ultrasound-based model	30	8	6	40	NR	NR	NR	NR	NR	NR	NR	NR
Wei et al. (34)	2023	Machine Learning	LR	Ultrasound&clinical model	52	2	16	71	NR	NR	NR	NR	52	33	16	40
Wen et al. (35)	2022	Machine Learning	LR	Ultrasound&clinical model	24	8	11	25	NR	NR	NR	NR	7	0	28	33
Wu et al. (36)	2024	Machine Learning	LR	Ultrasound&clinical model	22	6	5	29	NR	NR	NR	NR	25	15	2	20
Park et al. (37)	2020	Machine Learning	LR	Ultrasound&clinical model	69	126	31	142	NR	NR	NR	NR	NR	NR	NR	NR
Yan et al. (38)	2023	Machine Learning	LR	Ultrasound-based model	42	4	3	34	NR	NR	NR	NR	NR	NR	NR	NR
Yao et al. (39)	2022	Deep Learning	DCNN	Ultrasound&clinical model	451	43	102	307	NR	NR	NR	NR	NR	NR	NR	NR
Yu et al. (40)	2020	Deep Learning	TLR	Ultrasound&clinical model	379	140	24	470	180,207	17,74	37,11	134,221	NR	NR	NR	NR
Yuan et al. (41)	2024	Deep Learning	CNN	Ultrasound-based model	107	6	14	79	NR	NR	NR	NR	104	16	17	69
Zhang et al. (42)	2025	Deep Learning	CNN	Ultrasound-based model	37	5	10	31	44	13	3	35	28	17	19	31
Zhang et al. (43)	2023	Machine Learning	LR	Ultrasound&clinical model	19	9	16	150	NR	NR	NR	NR	NR	NR	NR	NR
Zhou et al. (44)	2022	Machine Learning	LR	Ultrasound&clinical model	92	40	21	173	NR	NR	NR	NR	15	16	98	197
Zhu et al. (45)	2023	Machine Learning	RF	Ultrasound&clinical model	26	17	12	63	NR	NR	NR	NR	NR	NR	NR	NR

TP, true positive; TN, true negative; FP, false positive; FN, false negative; NR, not report; LDA, linear discriminant analysis; LR, logistic regression; CNN, convolutional neural network; SVM, support vector machine; XGBoost, eXtreme gradient boosting; Fisher, Fisher's stepwise discriminant analysis; DCNN, deep convolutional neural network; TLR, transfer learning radiomics; RF, random forest.

**Figure 2:**
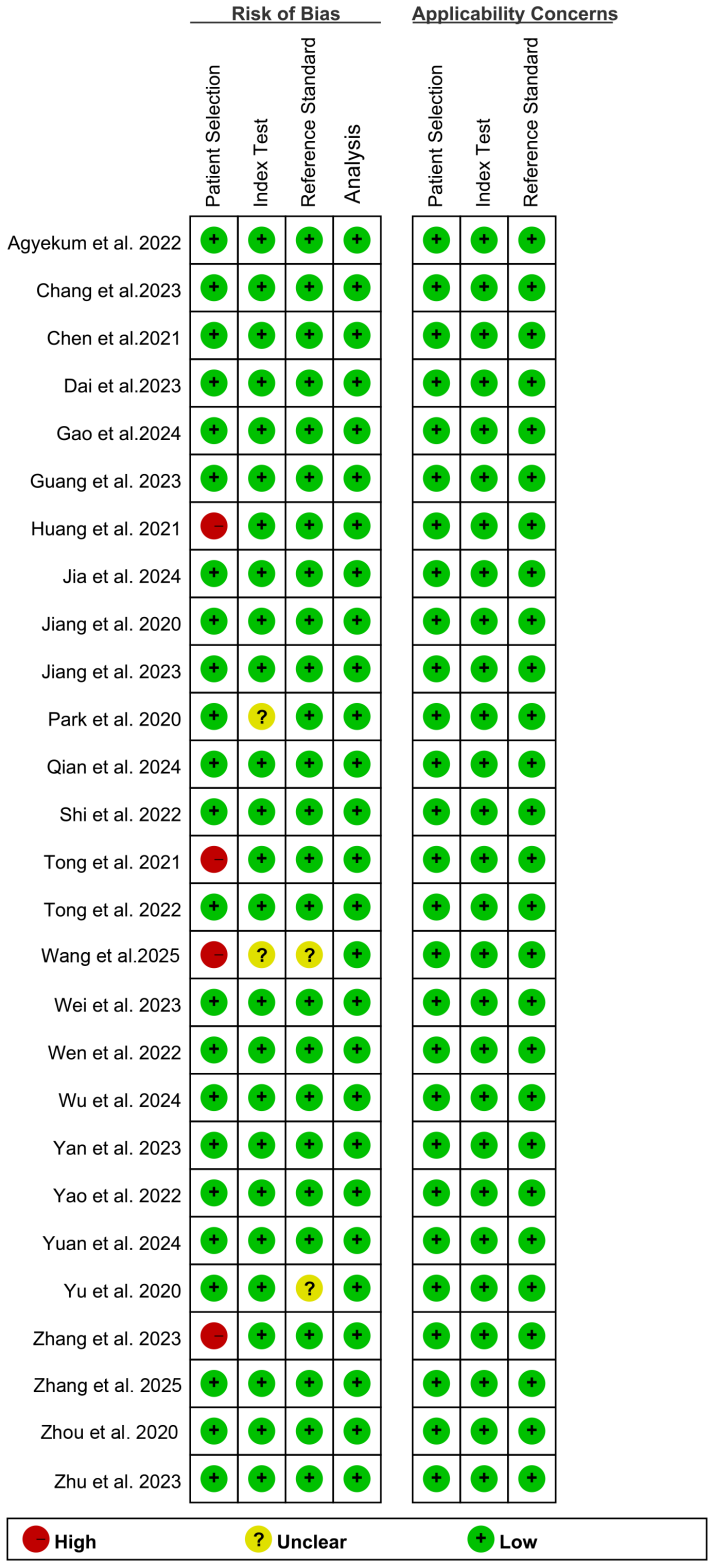
Risk of bias and applicability concerns of the included studies using the Quality Assessment of Diagnostic Performance Studies (QUADAS)-2 Revised tool.

According to the QUADAS-2-Revised tool, the risk of bias for each study is shown in **Figure 2**. For the bias assessment regarding Patient Selection, 4 studies were rated as “high risk” due to inappropriate exclusion. For the Index Test, 2 studies were rated as “unclear” because it was uncertain whether the AI model provided important training information. Regarding the Reference Standard, 2 studies were rated as “unclear” because it was uncertain whether the pathologists were aware of the pathology results in the final diagnosis. Overall, the quality assessment indicates that the quality of the included studies is acceptable.

### Diagnostic performance of internal validation set for AI and US physicians in predicting CLNM of PTC

**Figure 3:**
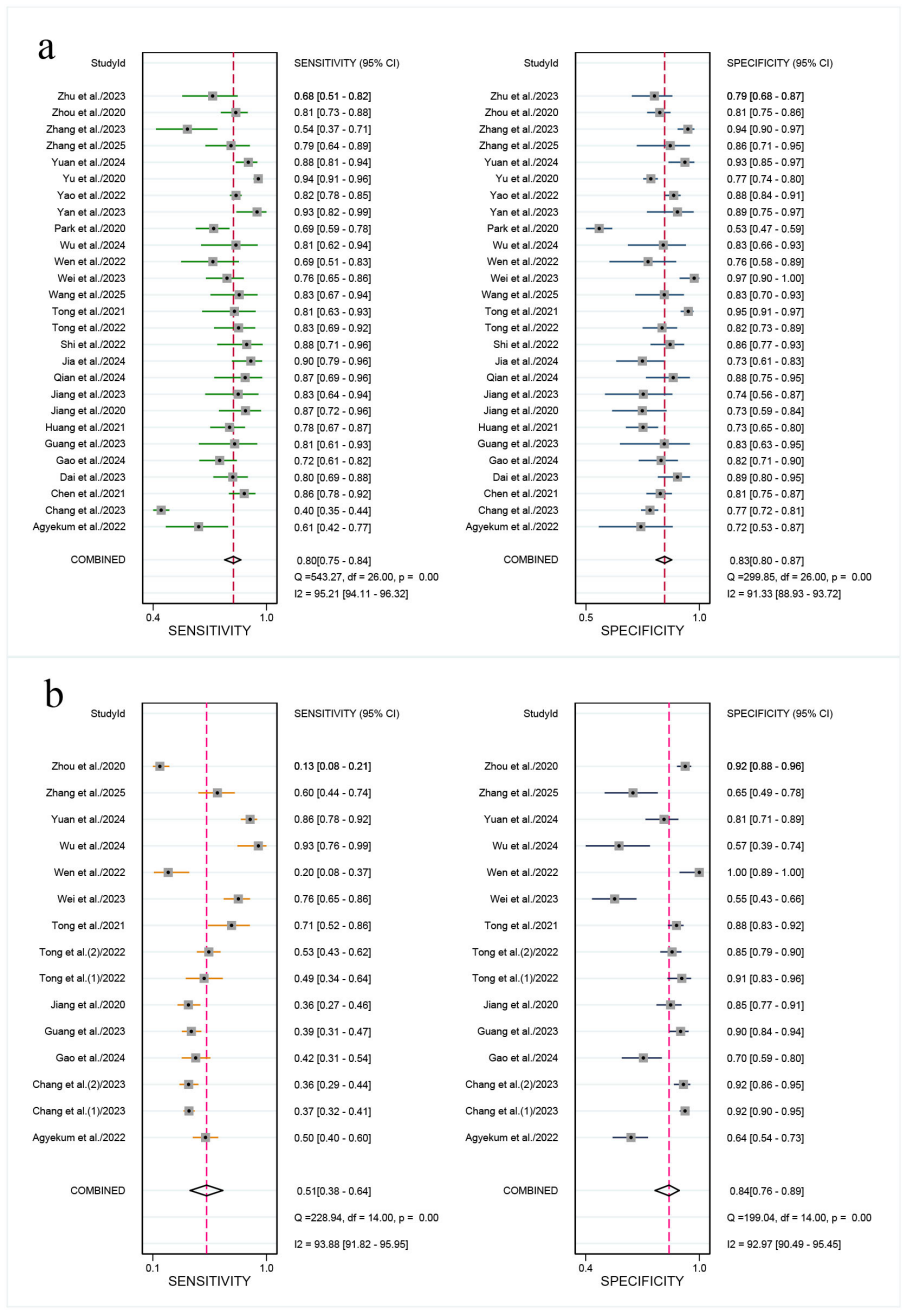
Forest plots showing the combined sensitivity and specificity of ultrasonography-based artificial intelligence in patients with cervical lymph node metastasis from papillary thyroid carcinoma: internal validation set **(a)** and ultrasound physicians **(b)**. Squares represent the sensitivity and specificity in each study, while horizontal bars indicate the 95% confidence intervals.

**Figure 4:**
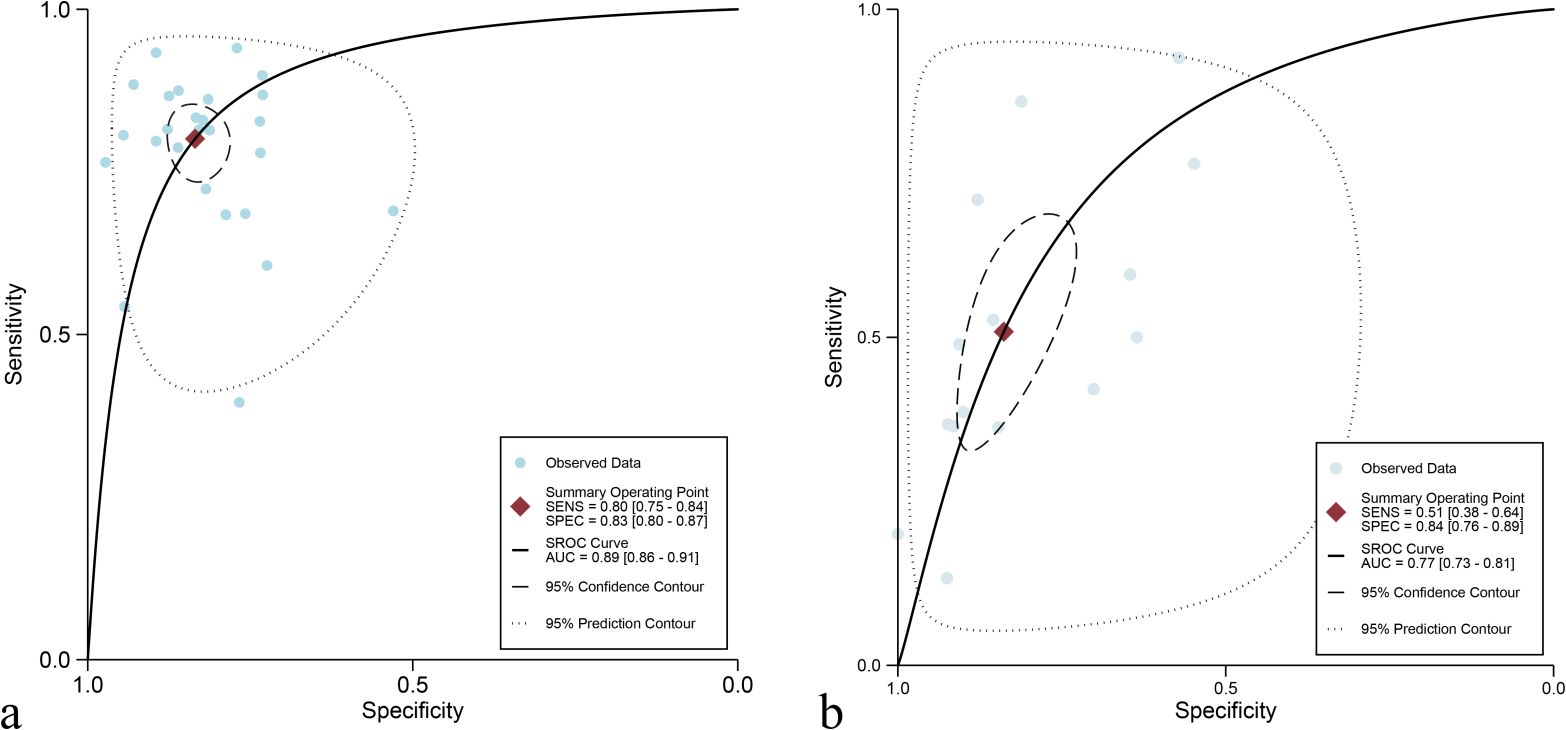
Summary receiver operating characteristic (SROC) curves for diagnosing cervical lymph node metastasis in papillary thyroid carcinoma: ultrasonography-based artificial intelligence on the internal validation set **(a)** and ultrasound physicians **(b)**.

**Figure 5:**
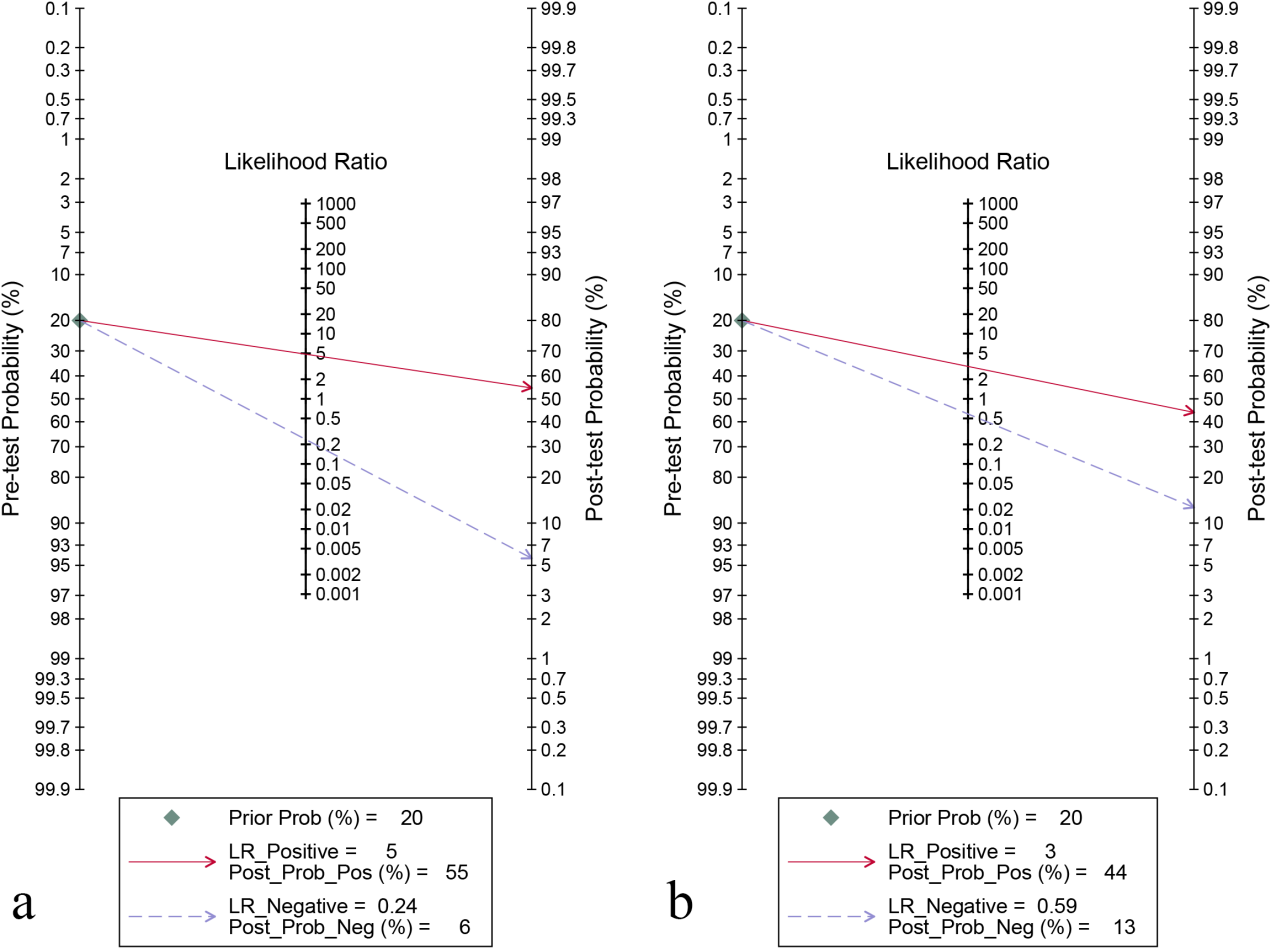
Fagan’s nomogram for diagnosing cervical lymph node metastasis in papillary thyroid carcinoma: ultrasonography-based artificial intelligence on the internal validation set **(a)** and ultrasound physicians **(b)**.

For the internal validation set, the sensitivity of AI in detecting CLNM of PTC was 0.80 (95% CI: 0.75-0.84) and the specificity was 0.83 (95% CI: 0.80-0.87) (**Figure 3a**), with an AUC of 0.89 (95% CI: 0.86-0.91) (**Figure 4a**). Using a pre-test probability of 20%, the Fagan nomogram indicated a positive likelihood ratio of 55% and a negative likelihood ratio of 6% (**Figure 5a**). For US physicians, the sensitivity for detecting CLNM of PTC was 0.51 (95% CI: 0.38-0.64) and the specificity was 0.84 (95% CI: 0.76-0.89) (**Figure 3b**), with an AUC of 0.77 (95% CI: 0.73-0.81) (**Figure 4b**). Using a 20% pre-test probability, the Fagan nomogram showed a positive likelihood ratio of 44% and a negative likelihood ratio of 13% (**Figure 5b**). The Z-test indicated that AI had significantly higher sensitivity and AUC values (*P* < 0.001), while there was no significant difference in specificity (*P* = 0.79).

**Figure 6:**
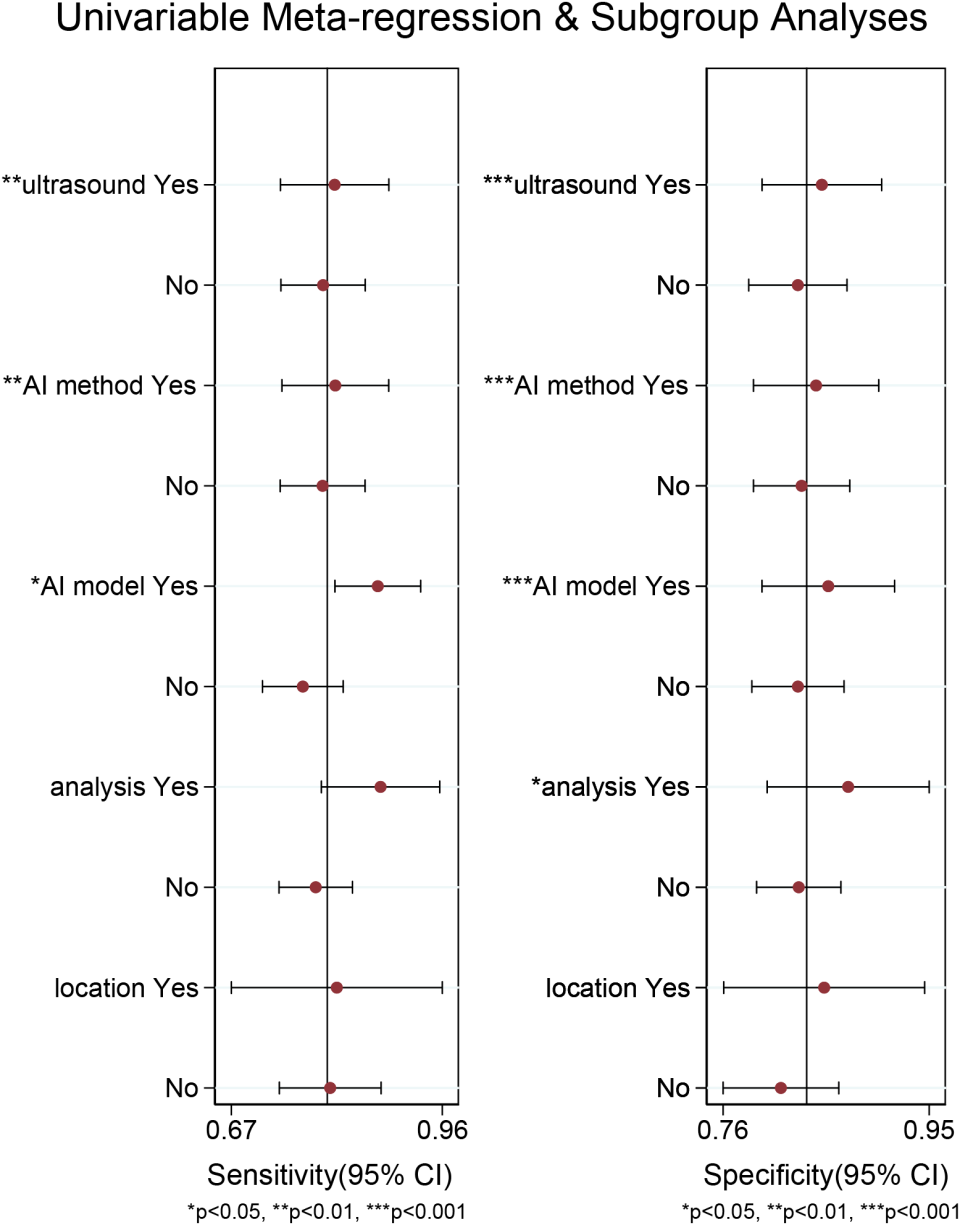
Meta-regression analysis of the internal validation set for diagnosing cervical lymph node metastasis in papillary thyroid carcinoma.

For the internal validation set, both sensitivity (I^2^ = 95.21%) and specificity (I^2^ = 91.33%) exhibited high heterogeneity. Meta-regression analysis indicated that the heterogeneity was primarily attributed to US techniques (sensitivity *P* < 0.01, specificity *P* < 0.001), AI methods (sensitivity *P* < 0.01, specificity *P* < 0.001), AI models (sensitivity *P* < 0.05, specificity *P* < 0.001), and types of data analysis (specificity *P* < 0.05) (**Figure 6**).

### Diagnostic performance of external validation sets for AI in predicting CLNM of PTC

For the external validation set, the sensitivity for detecting CLNM of PTC was 0.77 (95% CI: 0.49-0.92) and the specificity was 0.82 (95% CI: 0.75-0.88) (**Supplementary Figure S1**), with an AUC of 0.86 (95% CI: 0.83-0.89) (**Supplementary Figure S2**). Using a pre-test probability of 20%, the Fagan nomogram indicated a positive likelihood ratio of 52% and a negative likelihood ratio of 6% (**Supplementary Figure S3**).

### Diagnostic performance of subgroup analysis for AI in predicting CLNM of PTC

In the subgroups of ultrasound techniques, B-mode US had a sensitivity of 0.81 (95% CI: 0.76-0.86) and Multimodal US 0.78 (95% CI: 0.69-0.85), with no significant difference (*P* = 0.49). The specificity was 0.82 (95% CI: 0.76-0.86) for B-mode and 0.86 (95% CI: 0.80-0.91) for Multimodal US, also showing no significant difference (*P* = 0.23) (**Table 3**).

**Table 3:** Subgroup analysis of cervical lymph node metastasis of papillary thyroid carcinoma of internal validation set.

Subgroup	Studies, n	Sensitivity (95%CI)	Subgroup difference *P*-value	Specificity (95%CI)	Subgroup difference *P*-value
Ultrasound techniques			0.49		0.23
B-mode ultrasound	17	0.81 (0.75-0.86)		0.82 (0.76-0.86)	
Multimodal ultrasound	10	0.78 (0.69-0.85)		0.86 (0.80-0.91)	
AI method			0.19		0.91
Deep learning	9	0.84 (0.76-0.89)		0.83 (0.76-0.88)	
Machine learning	18	0.78 (0.71 - 0.84)		0.83 (0.78 - 0.88)	
AI model			<0.001		0.93
Ultrasound-based model	8	0.88 (0.82-0.92)		0.83 (0.76-0.89)	
Ultrasound&clinical model	19	0.76 (0.70-0.81)		0.83 (0.78-0.87)	
Analysis			0.12		0.29
Patient-based	23	0.79 (0.73-0.83)		0.82 (0.78-0.86)	
Lesion-based	4	0.87 (0.77-0.93)		0.87 (0.78-0.93)	
Location of cervical lymph node metastasis			0.49		0.04
Central	14	0.82 (0.76-0.87)		0.80 (0.74-0.86)	
Lateral	3	0.80 (0.64-0.90)		0.91 (0.84-0.95)	

For AI methods, the sensitivity was 0.84 (95% CI: 0.76-0.89) for deep learning and 0.78 (95% CI: 0.71-0.84) for machine learning, with no significant difference (*P* = 0.19). Both methods had a specificity of 0.83 (95% CI: 0.76-0.88), with no significant difference (*P* = 0.91) (**Table 3**).

Regarding AI models, the sensitivity of the US-based model was 0.88 (95% CI: 0.82-0.92) compared to 0.76 (95% CI: 0.70-0.81) for the US & clinical model, showing a significant difference (*P* < 0.001). Both models exhibited a specificity of 0.83 (95% CI: 0.76-0.89), with no significant difference (*P* = 0.93) (**Table 3**).

For data analysis types, patient-based sensitivity was 0.79 (95% CI: 0.73-0.83) and lesion-based was 0.87 (95% CI: 0.77-0.93), with no significant difference (*P* = 0.12). Specificity was 0.82 (95% CI: 0.78-0.86) for patient-based and 0.87 (95% CI: 0.78-0.93) for lesion-based, also with no significant difference (*P* = 0.29) (**Table 3**).

In terms of CLNM locations, sensitivity was 0.82 (95% CI: 0.76-0.87) for central and 0.80 (95% CI: 0.64-0.90) for lateral locations, showing no significant difference (*P* = 0.49). However, specificity was 0.80 (95% CI: 0.74-0.86) for central and 0.91 (95% CI: 0.84-0.95) for lateral, indicating a significant difference (*P* < 0.05) (**Table 3**).

### Publication bias

Deeks’ funnel plot asymmetry test indicated no significant publication bias for the internal validation set of AI and US physicians (*P* = 0.47, 0.86) (**Supplementary Figure S4-S5**). For the external validation set, no significant publication bias was observed either (*P* = 0.49) (**Supplementary Figure S6**).

## Discussion

Our meta-analysis revealed that AI-based ultrasonography demonstrated superior performance compared to human US physicians in detecting CLNM in patients with PTC. Specifically, AI achieved higher sensitivity, specificity, and AUC values. This enhanced diagnostic performance is largely attributable to AI’s ability to process large and complex datasets, extracting subtle, high-dimensional features that may be imperceptible to human observers (46). AI can integrate multiple imaging characteristics—such as texture, density, and signal intensity—into predictive models, thereby improving diagnostic precision (47). Internal validation datasets, which are typically more homogeneous and closely aligned with the training data, tend to yield better algorithm performance due to their consistency in imaging protocols and patient characteristics (48). Conversely, external validation datasets often introduce greater heterogeneity due to the imaging techniques, equipment, and patient populations (48). Interestingly, our findings demonstrate remarkable generalizability of the AI models, with the AUC decreasing only marginally from 0.89 in internal validation to 0.86 in external validation. The lower sensitivity and AUC observed among US physicians underscores the operator-dependent nature of traditional ultrasonography and the inherent limitations of qualitative or semi-quantitative assessments. These findings further highlight the potential of AI to standardize diagnostic processes and improve accuracy in clinical practice.

It’s worth noting that our meta-analysis revealed no statistically significant differences in sensitivity (*P* = 0.19) or specificity (*P* = 0.91) between deep learning and machine learning methods. The sensitivity of deep learning and machine learning was 0.84 and 0.78, respectively, while both methods demonstrated a same specificity of 0.83. The comparable diagnostic performance may be explained by their shared reliance on advanced algorithmic frameworks capable of identifying critical imaging features relevant to CLNM prediction (49). Both approaches employ supervised learning techniques to analyze structured imaging data, enabling the detection of patterns such as texture, density, and morphological changes in lymph nodes (50). Deep learning, particularly CNN, has the advantage of automated feature extraction directly from raw data. In contrast, machine learning often relies on handcrafted features derived from expert knowledge (50). However, in this context, the imaging datasets used in the included studies may have been sufficiently optimized, with robust feature engineering for machine learning models, thereby reducing the performance gap between the two methods.

Another finding is that the results demonstrated a statistically significant difference in sensitivity between the US-based model and the US & clinical model for predicting CLNM of PTC patients, with sensitivities of 0.88 and 0.76 (*P* < 0.001). The higher sensitivity of the US-based model may be attributed to its exclusive reliance on ultrasound imaging features, which are directly associated with structural and morphological changes in lymph nodes, such as size, echogenicity, and vascularity—key indicators for detecting CLNM (51). In contrast, the US & clinical model integrates additional clinical variables, such as patient demographics and laboratory findings, which may not be as strongly correlated with CLNM. These variables could introduce irrelevant or conflicting information, potentially diluting the predictive strength of the imaging features and resulting in lower sensitivity (51).

This meta-analysis also showed no statistically significant difference in sensitivity between the central and lateral locations of CLNM. However, specificity was significantly higher for the lateral lymph nodes (0.91) compared to the central lymph nodes (0.80; *P* < 0.05). The superior specificity for the lateral location may be attributed to the distinct anatomical and imaging characteristics of lateral lymph nodes. These nodes are typically larger, more superficial, and easier to visualize using ultrasonography (52). They also tend to exhibit clearer morphological changes, such as irregular margins, loss of the hilum, or abnormal vascularity, which facilitate differentiation from benign lymph nodes (52). In contrast, central lymph nodes are situated in a more anatomically complex region, often surrounded by structures such as the thyroid gland, trachea, and blood vessels. This complexity can obscure visualization on ultrasonography and result in overlapping features between metastatic and benign nodes, thereby reducing diagnostic specificity (53).

Previous meta-analyses have provided valuable insights into the diagnostic performance of various imaging modalities for LNM in thyroid cancer. For instance, the 2023 meta-analysis by HajiEsmailPoor et al. evaluated 25 studies assessing the performance of CT, US, and MRI-based radiomics for predicting LNM in PTC (54). Their results indicated that US outperformed CT and MRI, with a sensitivity of 0.77 and a specificity of 0.79. Our study, focusing exclusively on AI-based models using US for predicting CLNM of PTC, revealed even higher diagnostic performance, with pooled sensitivity and specificity of 0.80 and 0.83. This improvement may be attributed to the advanced analytical capabilities of AI, as incorporating more US-based AI studies allows it to extract and analyze subtle imaging features beyond human perception. Furthermore, unlike previous studies, our study is the first meta-analysis to focus on US-based AI models and their relative diagnostic performance compared to US physicians for CLNM of PTC, offering a more targeted and comprehensive result (55).

In comparison to the 2024 meta-analysis by Zhang et al., which examined radiomics-based US models for LNM in thyroid cancer, our study yielded slightly lower diagnostic performance (56). This discrepancy may be explained by differences in study populations, as Zhang et al. included various thyroid cancers (including PTC), while our analysis was restricted to PTC cases. It is important to notethat our study introduced two significant innovations: the first direct comparison of AI models with US physicians, highlighting the potential clinical advantages of AI, and a subgroup analysis evaluating diagnostic performance using internal and external validation datasets. These advancements provide critical evidence for the practical application of AI in clinical settings and address limitations in prior meta-analyses.

This study highlights that significant heterogeneity among the included studies may have impacted the overall sensitivity and specificity of AI in internal test datasets. Meta-regression analysis identified US techniques, AI methods, and AI models as potential sources of heterogeneity affecting sensitivity. The potential source of heterogeneity for specificity were the types of data analysis. Despite this heterogeneity, the findings demonstrate that US-based AI achieves high diagnostic performance for predicting CLNM of PTC across both internal and external validation datasets, surpassing the diagnostic performance of US physicians. This suggests that AI has the potential to alleviate the workload of clinical practitioners, reduce misdiagnoses and missed diagnoses, and prevent adverse outcomes associated with the disease. The integration of US-based AI tools into primary care settings, such as general practice, could support early detection and timely management of PTC. Moreover, US-based AI has the potential to enhance screening efficiency, particularly in resource-constrained or remote areas where access to specialized expertise is limited. In the future, US-based AI systems could serve as valuable tools to assist US physicians in making more accurate diagnoses.

However, while diagnostic performance is crucial, cost-effectiveness is an equally important consideration when introducing new technologies into routine clinical practice. AI’s diagnostic potential raises ethical and operational concerns, including tensions between algorithmic efficiency and clinician autonomy due to opaque “black-box” systems, as well as bias risks from non-representative training data that may worsen health inequities (57). Mitigation strategies could involve adopting explainable AI to clarify decisions, implementing bias-checking validation protocols, and establishing oversight-focused regulatory policies with hybrid human-AI workflows to balance innovation with accountability (58). Notably, this study did not identify any research evaluating the cost-effectiveness of AI in diagnosing CLNM of PTC, underscoring a critical gap that future investigations should address.

The limitations of this study should be acknowledged. First, there is a lack of external validation among the included studies, with only four out of 27 studies performing external validation. External validation is crucial because overfitting is a common issue in AI training (48). Second, most of the included studies were retrospective in design, which may introduce potential biases. Well-designed prospective studies are necessary to confirm the findings of this meta-analysis and ensure their robustness. Third, three studies used non-pathology-based reference standards, which could introduce bias in the evaluation of diagnostic performance. Fourth, this study only included English-language literature, a decision primarily driven by pragmatic considerations of accessibility. However, it may bring potential publication bias. Future research should adopt more standardized and consistent pathology-based reference standards to ensure accuracy and reliability.

## Conclusion

US-based AI demonstrates higher diagnostic performance than clinicians. However, the high heterogeneity among studies limits the strength of these findings, necessitating further investigation of external validation datasets to confirm the results and assess their practical clinical value.

## Data Availability

The original contributions presented in the study are included in the article/**Supplementary Material**. Further inquiries can be directed to the corresponding author.
