# Supplementary material for: Ultrasound-based artificial intelligence for predicting cervical lymph node metastasis in papillary thyroid cancer: a systematic review and meta-analysis
Source: Front Endocrinol (Lausanne). 2025 Jun 10;16:1570811. doi: 10.3389/fendo.2025.1570811 (PMC12185295; doi:10.3389/fendo.2025.1570811)

Supplementary Table 1 Search strategy in PubMed, Embase and Web of Science.

| Database | Search strategy |
| --- | --- |
| PubMed | ("Artificial Intelligence"[Mesh] OR "Machine Learning"[Mesh] OR "Deep Learning"[Mesh] OR "Radiomics"[Mesh] OR "Artificial Intelligence"[Title/Abstract] OR "AI"[Title/Abstract] OR "Machine Learning"[Title/Abstract] OR "Deep Learning"[Title/Abstract] OR “Radiomic”[Title/Abstract]) AND ("Lymphatic Metastasis"[Mesh] OR "Lymph Node Metastasis"[Title/Abstract] OR "LNM"[Title/Abstract] OR "node Metastasis" [Title/Abstract]) AND ("Thyroid Neoplasms"[Mesh] OR "Thyroid Neoplasm"[Title/Abstract] OR "Neoplasms, Thyroid"[Title/Abstract] OR "Thyroid Carcinoma"[Title/Abstract] OR "Carcinoma, Thyroid"[Title/Abstract] OR "Carcinomas, Thyroid"[Title/Abstract] OR "Thyroid Carcinomas"[Title/Abstract] OR "Cancer of Thyroid"[Title/Abstract] OR "Thyroid Cancers"[Title/Abstract] OR "Thyroid Cancer"[Title/Abstract] OR "Cancer, Thyroid"[Title/Abstract] OR "Cancers, Thyroid"[Title/Abstract] OR "Cancer of the Thyroid"[Title/Abstract] OR "Thyroid Adenomas"[Title/Abstract] OR “thyroid tumor”[Title/Abstract] OR “thyroid neoplasm”[Title/Abstract] OR “thyroid carcinoma”[Title/Abstract]) |
| Embase | ('artificial intelligence'/exp OR 'machine learning'/exp OR 'deep learning'/exp OR 'radiomics'/exp OR ‘Artificial Intelligence’:ab,ti OR ‘AI’:ab,ti OR ‘Machine Learning’:ab,ti OR ‘Deep Learning’:ab,ti) AND ('thyroid carcinoma'/exp OR 'thyroid tumor'/exp OR 'Thyroid Neoplasm':ab,ti OR 'Neoplasms, Thyroid':ab,ti OR 'Thyroid Carcinoma':ab,ti OR 'Carcinoma, Thyroid':ab,ti OR 'Carcinomas, Thyroid':ab,ti OR 'Thyroid Carcinomas':ab,ti OR 'Cancer of Thyroid':ab,ti OR 'Thyroid Cancers':ab,ti OR 'Thyroid Cancer':ab,ti OR 'Cancer, Thyroid':ab,ti OR 'Cancers, Thyroid':ab,ti OR 'Cancer of the Thyroid':ab,ti OR 'Thyroid Adenomas':ab,ti) AND ('lymph node metastasis'/exp OR 'Lymph Node Metastasis':ab,ti OR 'LNM':ab,ti OR 'node Metastasis':ab,ti) |
| Web of Science | ((TS=(“Neoplasm, Thyroid” OR “Thyroid Neoplasm” OR “Neoplasms, Thyroid” OR “Thyroid Carcinoma” OR “Carcinoma, Thyroid” OR “Carcinomas, Thyroid” OR “Thyroid Carcinomas” OR “Cancer of Thyroid” OR “Thyroid Cancers” OR “Thyroid Cancer” OR “Cancer, Thyroid” OR “Cancers, Thyroid” OR “Cancer of the Thyroid” OR “Thyroid Adenoma” OR “Adenoma, Thyroid” OR “Adenomas, Thyroid” OR “Thyroid Adenomas”)) AND TS=("Artificial Intelligence" OR "Machine Learning" OR "Deep Learning" OR "AI" OR "Machine Learning" OR "Deep Learning" OR “Radiomic”)) AND TS=("Lymphatic Metastasis" OR "Lymph Node Metastasis" OR " LNM" OR “node Metastasis”) |
| Cochrane Library | (“Neoplasm, Thyroid” OR “Thyroid Neoplasm” OR “Neoplasms, Thyroid” OR “Thyroid Carcinoma” OR “Carcinoma, Thyroid” OR “Carcinomas, Thyroid” OR “Thyroid Carcinomas” OR “Cancer of Thyroid” OR “Thyroid Cancers” OR “Thyroid Cancer” OR “Cancer, Thyroid” OR “Cancers, Thyroid” OR “Cancer of the Thyroid” OR “Thyroid Adenoma” OR “Adenoma, Thyroid” OR “Adenomas, Thyroid” OR “Thyroid Adenomas”) AND ("Artificial Intelligence" OR "Machine Learning" OR "Deep Learning" OR "Radiomics" OR “AI”) AND ("Lymphatic Metastasis" OR "Lymph Node" OR "LNM" OR "node" OR "Lymph Node metastasis") |

Supplementary Table 2 Revised QUADAS-2 tool for the included studies.

| Author | Risk of bias | | | |  | Applicability concerns | | |
| --- | --- | --- | --- | --- | --- | --- | --- | --- |
|  | Patient selection ^a^ | Index test ^b^ | Reference standard ^c^ | Analysis ^d^ |  | Patient selection ^e^ | Index test ^f^ | Reference standard ^g^ |
| Agyekum et al. 2022 | L | L | L | L |  | L | L | L |
| Chang et al.2023 | L | L | L | L |  | L | L | L |
| Chen et al.2021 | L | L | L | L |  | L | L | L |
| Dai et al.2023 | L | L | L | L |  | L | L | L |
| Gao et al.2024 | L | L | L | L |  | L | L | L |
| Guang et al. 2023 | L | L | L | L |  | L | L | L |
| Huang et al. 2021 | H | L | L | L |  | L | L | L |
| Jia et al. 2024 | L | L | L | L |  | L | L | L |
| Jiang et al. 2020 | L | L | L | L |  | L | L | L |
| Jiang et al. 2023 | L | L | L | L |  | L | L | L |
| Park et al. 2020 | L | U | L | L |  | L | L | L |
| Qian et al. 2024 | L | L | L | L |  | L | L | L |
| Shi et al. 2022 | L | L | L | L |  | L | L | L |
| Tong et al. 2021 | H | L | L | L |  | L | L | L |
| Tong et al. 2022 | L | L | L | L |  | L | L | L |
| Wang et al. 2025 | H | U | U | L |  | L | L | L |
| Wei et al. 2023 | L | L | L | L |  | L | L | L |
| Wen et al. 2022 | L | L | L | L |  | L | L | L |
| Wu et al. 2024 | L | L | L | L |  | L | L | L |
| Yan et al. 2023 | L | L | L | L |  | L | L | L |
| Yao et al. 2022 | L | L | L | L |  | L | L | L |
| Yuan et al. 2024 | L | L | L | L |  | L | L | L |
| Yu et al. 2020 | L | L | U | L |  | L | L | L |
| Zhang et al. 2023 | H | L | L | L |  | L | L | L |
| Zhang et al. 2025 | L | L | L | L |  | L | L | L |
| Zhou et al. 2020 | L | L | L | L |  | L | L | L |
| Zhu et al. 2023 | L | L | L | L |  | L | L | L |

L low; H high; U unclear.

a. Patient selection

• Low risk: No inappropriate exclusions.

• High risk: Inappropriate exclusions (e.g., excluding patients under 18, restricting to specific treatments/subtypes/timeframes).

• Unclear: Insufficient information to assess exclusions.

b. Index test

• Low risk: Detailed model training/validation processes provided or cited from a prior publication with full modification details.

• High risk: Only model name reported without key training details (e.g., algorithm unspecified).

• Unclear: Model name given but training process indeterminable.

c. Reference standard

• Low risk: Final diagnosis made blinded to AI results.

• High risk: AI results used to inform final diagnosis.

• Unclear: Blinding status unreported.

d. Analysis

• Low risk: All enrolled participants included in meta-analysis.

• High risk: Selective exclusion of participants/subgroups.

• Unclear: Inclusion criteria inadequately described.

e. Patient selection

• Low risk: Study population aligns with meta-analysis inclusion criteria.

• High risk: Study includes ineligible patients per meta-analysis criteria.

• Unclear: Population eligibility unclear.

f. Index test

• Low risk: AI definition matches meta-analysis criteria.

• High risk: AI definition partially deviates from criteria.

• Unclear: AI definition adequacy unverifiable.

g. Reference standard

• Low risk: Reference standard aligns with meta-analysis criteria.

• High risk: Reference standard inconsistently applied.

• Unclear: Reference standard details missing.

Supplementary Figure 1: Forest plots showing the combined sensitivity and specificity of ultrasonography-based artificial intelligence in patients with cervical lymph node metastasis from papillary thyroid carcinoma: external validation set


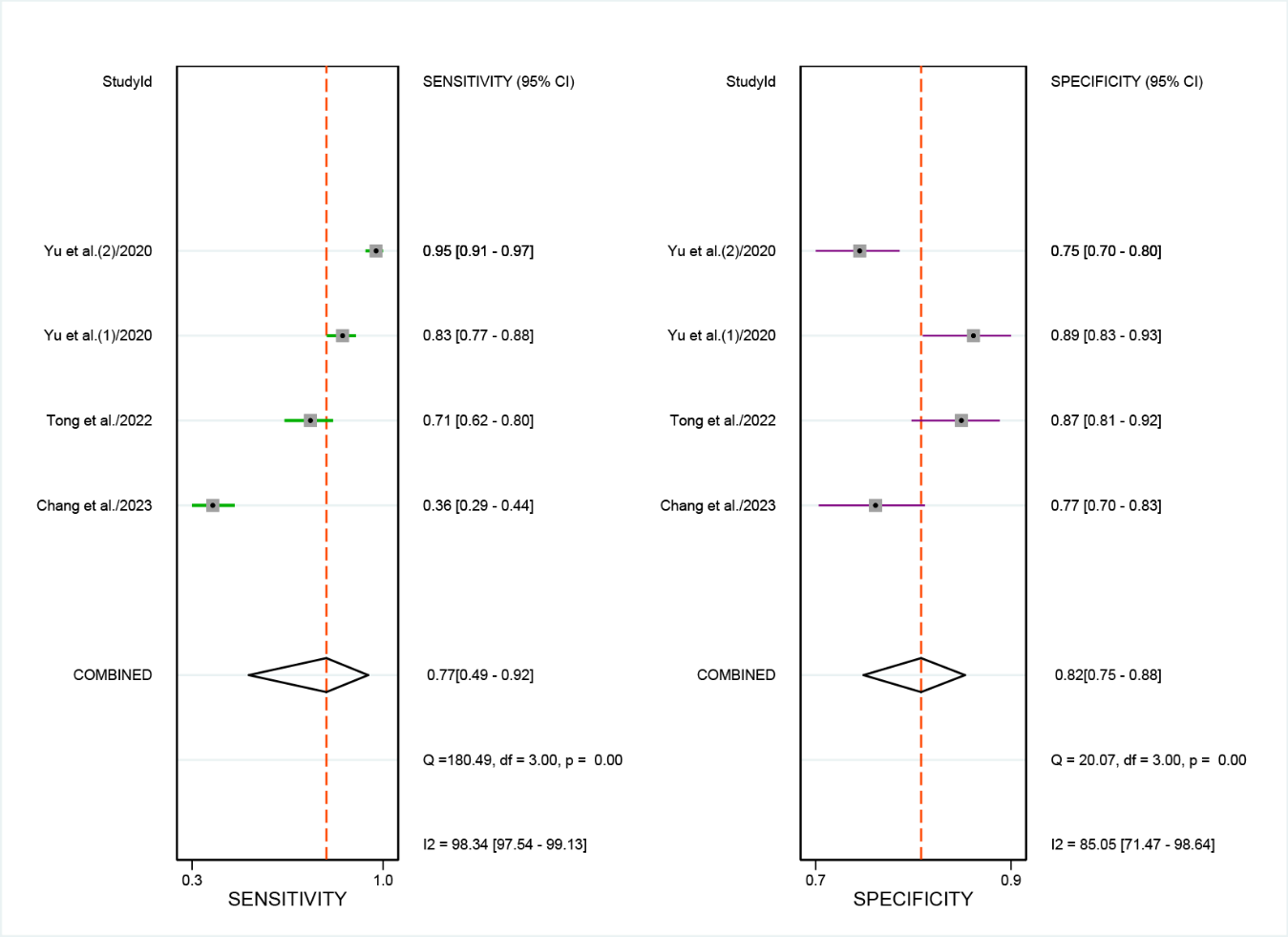


Supplementary Figure 2: Summary receiver operating characteristic (SROC) curves for diagnosing cervical lymph node metastasis in papillary thyroid carcinoma: ultrasonography-based artificial intelligence on the external validation set


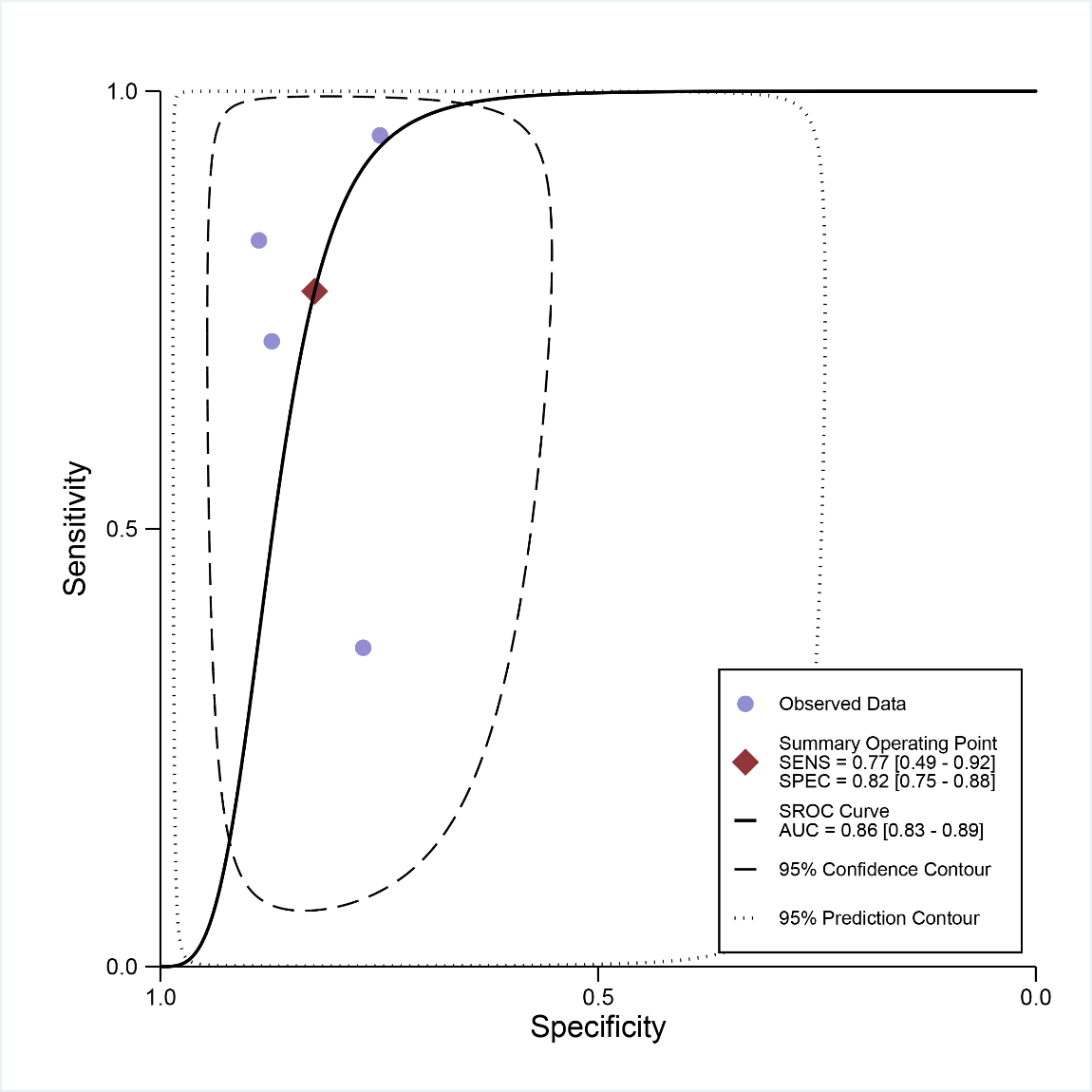


Supplementary Figure 3: Fagan's nomogram for diagnosing cervical lymph node metastasis in papillary thyroid carcinoma: ultrasonography-based artificial intelligence on the external validation set


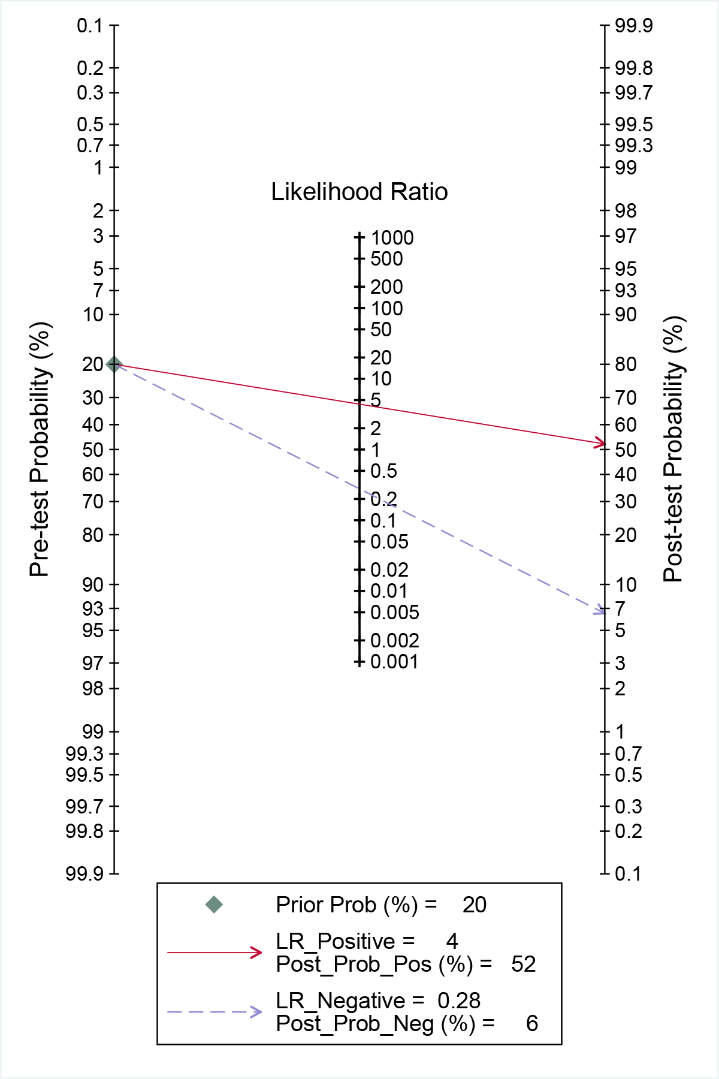


Supplementary Figure 4: Deeks' funnel plot for the internal validation set of artificial intelligence


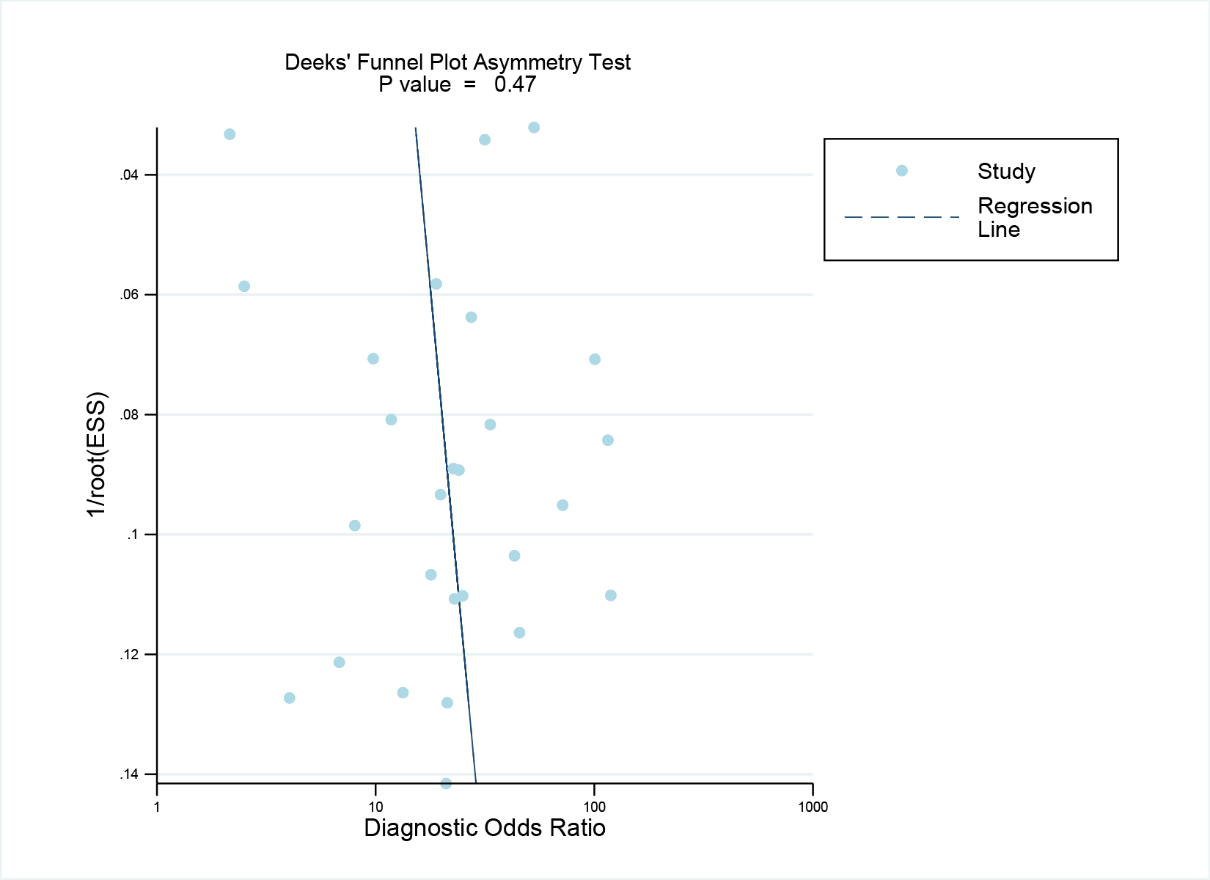


Supplementary Figure 5: Deeks' funnel plot for the ultrasonography physicians


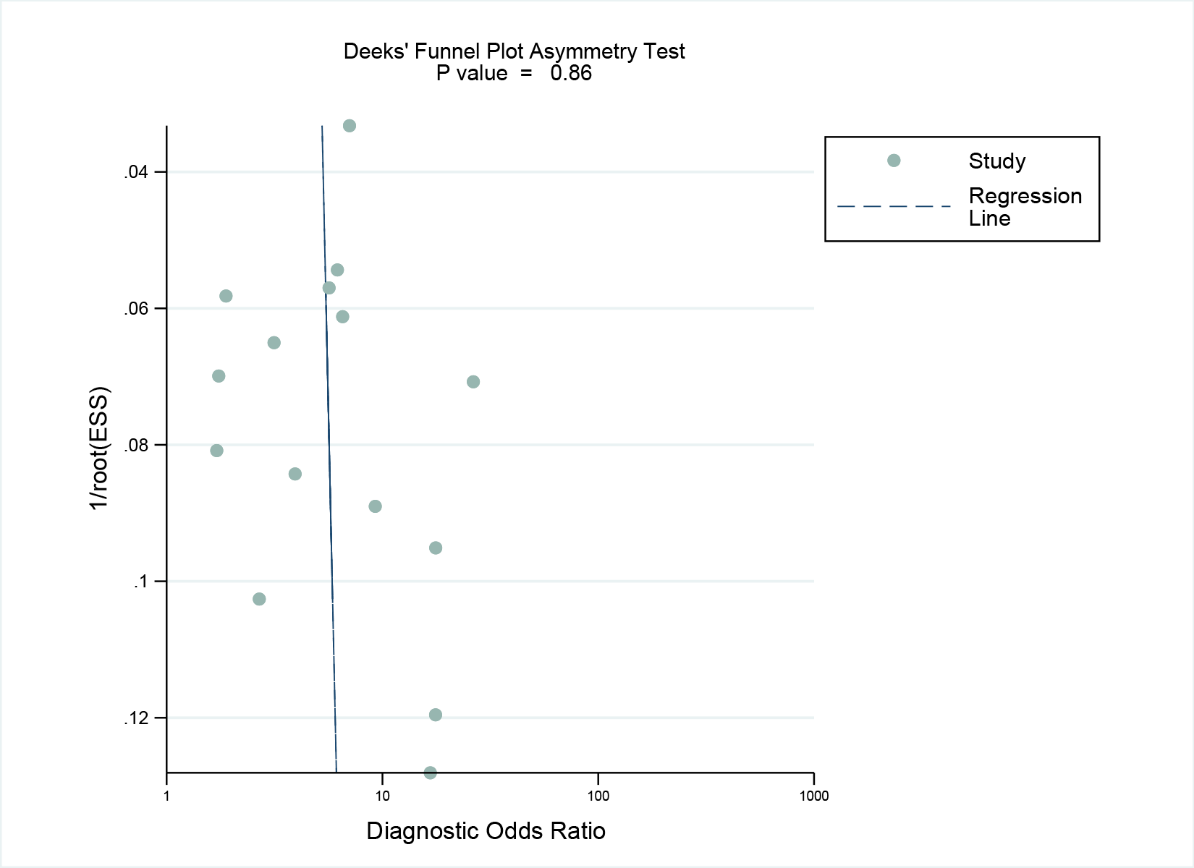


Supplementary Figure 6: Deeks' funnel plot for the external validation set of artificial intelligence


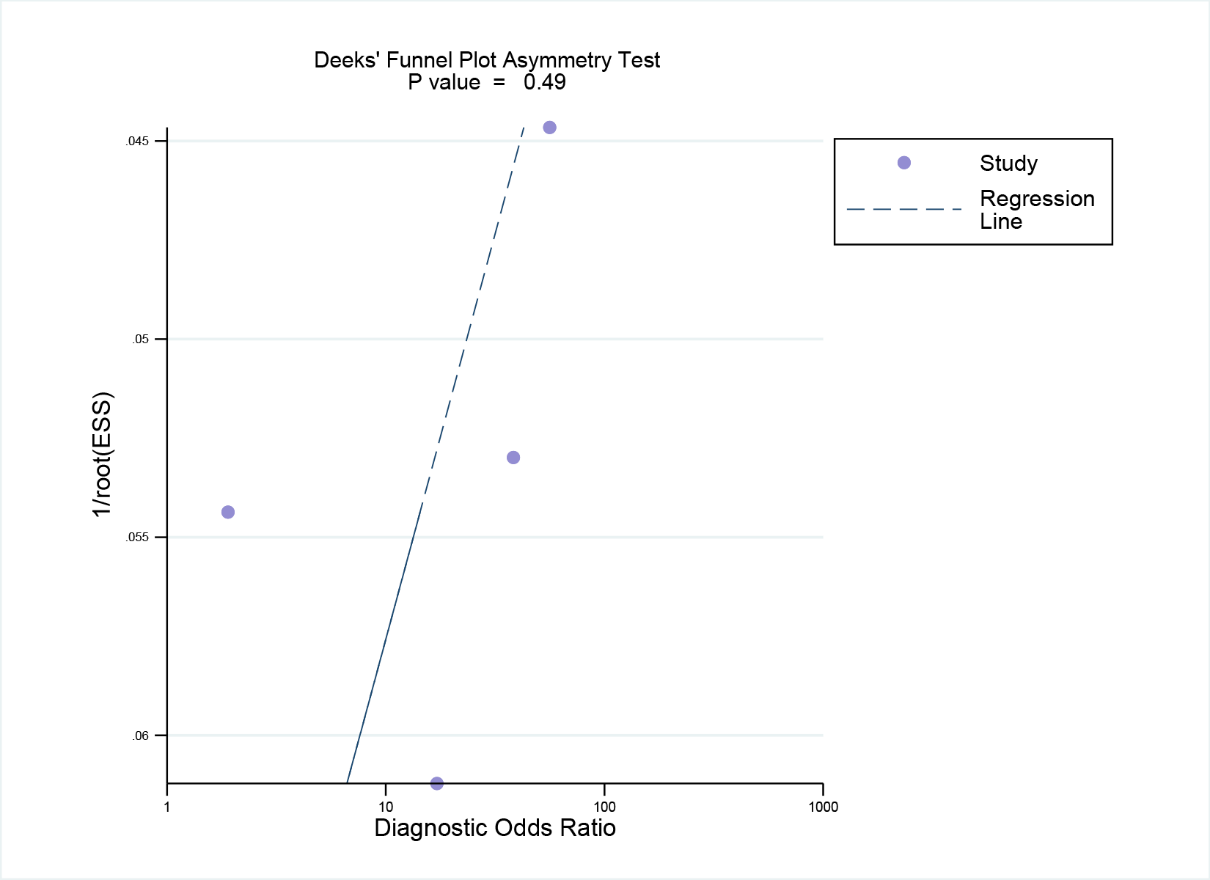

Supplement: Supplementary file 1 [file DataSheet1.docx]
